# Supplementary material for: Detection of Avian Leukosis Virus Subgroup J (ALV-J) Using RAA and CRISPR-Cas13a Combined with Fluorescence and Lateral Flow Assay
Source: Int J Mol Sci. 2024 Oct 7;25(19):10780. doi: 10.3390/ijms251910780 (PMC11476368; doi:10.3390/ijms251910780)
Supplement: Supplementary file 1 [file ijms-25-10780-s001.zip › ijms-3225021-supplementary.pdf]

Table S1. NCBI information of the strain

strain	BioSample accession
S1	KF218959.1
S2	KF218958.1
S3	KF201290.1
S4	PQ119500.1
S5	MN496126.1
S6	MN496122.1
S7	PQ010741.1
S8	MK829813.1
S9	MH023420.1
S10	MK104145.1
S11	MH669346.1
S12	KY379033.1
S13	DQ316908.1
S14	KP317566.1
S15	KP317565.1
S16	KM655822.1
S17	KM655820.1
S18	KJ631319.1
S19	KJ631317.1
